# Lessons from Plant–Microbe Interactions: Concepts for Improving Plant Nutrition and Stress Tolerance

**DOI:** 10.3390/ijms27219452

**Published:** 2026-10-23

**Authors:** Mahmoud W. Yaish, Axel Mithöfer

**Affiliations:** 1 Department of Biology, College of Sciences, Sultan Qaboos University, P.O. Box 36, Alkhoud, Muscat 123, Oman; 2 Research Group Plant Defense Physiology, Max-Planck-Institute for Chemical Ecology, Hans-Knöll-Straße 8, D-07745 Jena, Germany

**Keywords:** microbial consortia, PGPB biofertilizers, plant stress resilience, precision agriculture, rhizosphere microbiome

## Abstract

Plant-associated microorganisms, particularly plant growth-promoting bacteria (PGPB), enhance plant nutrition and stress tolerance and offer potential complementary approaches to conventional fertilization and crop protection. However, inconsistent field performance, ecological concerns associated with introducing non-native bacterial strains, and the need for formulations tailored to specific plant genotypes and soil conditions limit their agricultural application. These challenges have stimulated research into complementary strategies for promoting plant growth under favorable and stressful conditions. Here, we review advances and limitations of PGPB-based biofertilizers, focusing on multi-strain consortia, endophytic versus epiphytic microorganisms, and precision agriculture approaches integrating soil microbiome dynamics with plant-genotype-specific formulations. We also examine molecular mechanisms through which PGPB influence plant gene expression, stress signaling, nutrient acquisition, and rhizosphere communication. Building on these insights, we discuss strategies for using beneficial microbial mechanisms to improve abiotic stress tolerance and nutrient uptake. Integrating microbiome-informed biofertilizer development with genetic engineering, crop breeding, and precision agriculture could improve plant resilience. However, successful application requires functional validation and field testing under variable environmental conditions. Such approaches may reduce dependence on external agricultural inputs while improving crop productivity and sustainability. Together, these complementary strategies provide a framework for harnessing plant–microbe interactions to support climate-resilient agriculture and future food security worldwide.

## 1. Introduction

The global population is projected to reach 9.7 billion by 2050 and is expected to peak at approximately 10.3 billion in the mid-2080s, placing exceptional pressure on food systems and natural resources [1]. In addition, climate change-induced challenges such as longer drought periods, saline soils, or water scarcity disproportionately affect developing regions, including sub-Saharan Africa and the Middle East, where poverty, conflict, and import dependence remain high [2,3]. Moreover, efforts to meet the growing demand for crops in agriculture have relied heavily on chemical fertilizers, which may increase yields but also contribute to soil degradation, groundwater pollution, biodiversity loss, and greenhouse gas emissions, thereby undermining long-term food security [4,5].

Agricultural research has increasingly shifted toward sustainable alternatives, including biofertilizers prepared from PGPB. PGPB can enhance nutrient acquisition, stimulate plant growth, suppress phytopathogens, and support soil biodiversity while reducing dependence on chemical inputs [6,7]. A meta-analysis reported an average crop yield increase of 33.5% following microbial inoculation under field conditions, although responses varied with microbial inoculant species and crop type [8]. Another global meta-analysis found that biofertilization improved phosphorus- and nitrogen-use efficiency by 7.5 and 5.8 kg yield per kg of applied P and N, respectively [9]. These benefits may also extend to reducing the environmental impacts of fertilization, with microbial inoculation reported to decrease N_2_O emissions and NH_3_ volatilization from fertilized soils by 29.8% and 24.9%, respectively [10]. The potential of PGPB is highly important in resource-limited, climate-vulnerable regions, where locally adapted microbial strains might enhance both crop resilience and productivity [11]. Numerous studies and reviews published in recent years have reported beneficial effects of plant-associated microorganisms on plant growth, nutrition, and stress responses [12,13]. However, potential limitations and context-dependent outcomes have received comparatively less attention. For example, effective implementation of PGPB remains constrained by variable performance, complex rhizosphere interactions, and the need for sustained investment in research, technology transfer, and agronomic capacity building [14,15]. Despite extensive evidence demonstrating that plant-associated microbes can enhance stress tolerance under controlled laboratory conditions, a major limitation is the inconsistent reproducibility of microbial inoculants in field environments [16]. Therefore, evidence obtained under controlled laboratory or greenhouse conditions should be distinguished from field-validated responses, where microbial establishment, persistence, and plant performance are influenced by substantially greater environmental complexity. Accordingly, positive results obtained under controlled conditions should be interpreted as evidence of potential efficacy rather than confirmation of consistent agronomic benefits under field conditions. Multiple experimental studies and reviews have concluded that the performance of microbial inoculants is highly context-dependent, with benefits often attenuated or even lost under natural conditions [17]. Environmental fluctuations, including the legacy of the soil microbiome, competition with resident microbial communities, plant genotypes, and fluctuating abiotic factors such as moisture and nutrient availability, can significantly influence the establishment, persistence, and host responsiveness of PGPB, thereby highlighting the limitations of transferring results from laboratory and greenhouse experiments to field conditions [18]. Additionally, concerns have been raised that introducing new bacterial strains into biofertilizers may disrupt the native microbial community [19], making biofertilizer applications a potentially sensitive intervention in agriculture.

**Figure 1:**
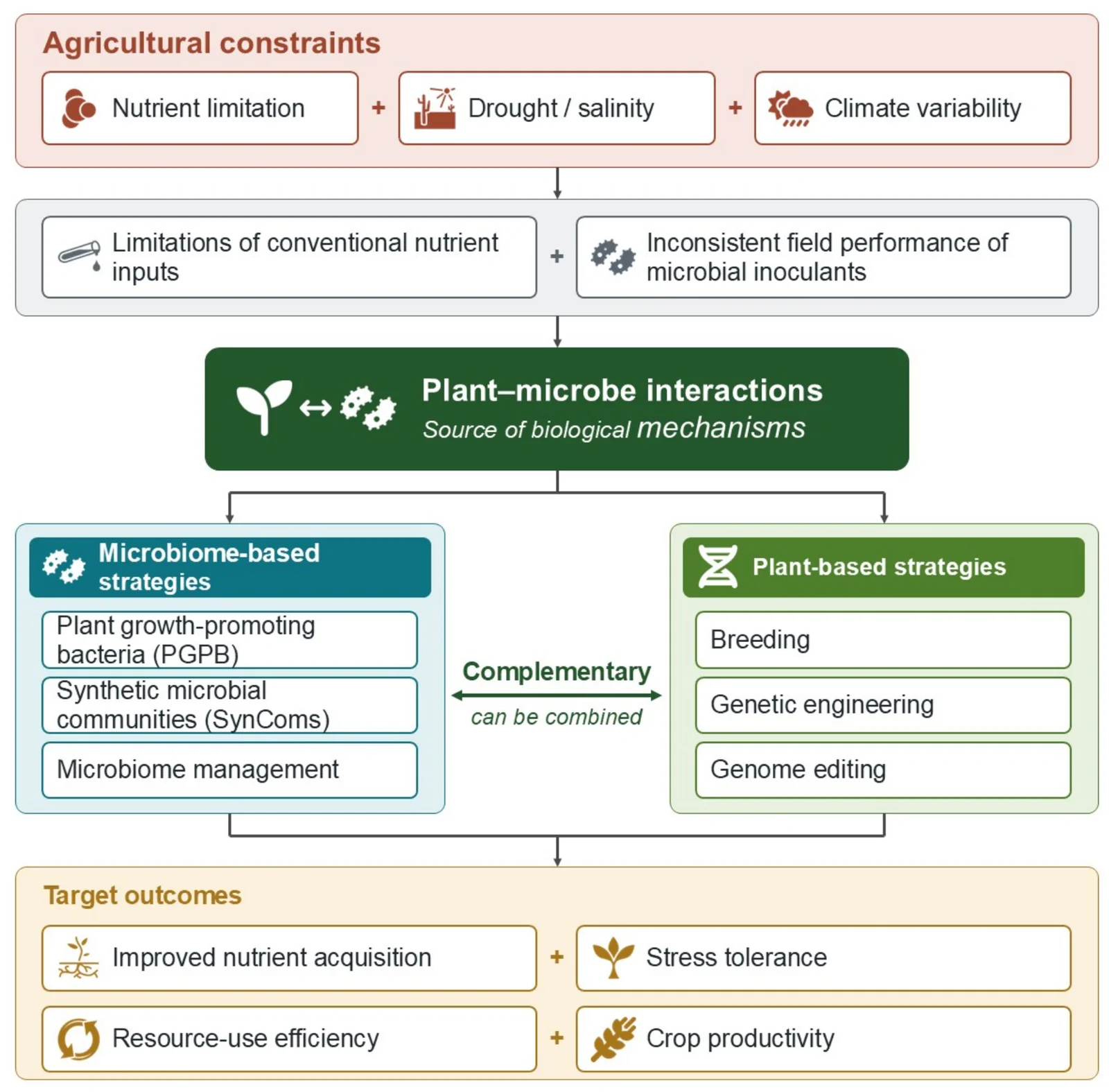
The translation of plant–microbe interactions into strategies for improving nutrient supply, stress tolerance, and productivity of crops within the framework of sustainable agriculture.

The major challenges addressed in this review and the complementary microbiome- and plant-based strategies proposed to improve plant nutrition and stress resilience are summarized in Figure 1. This review article highlights recent developments in the use of PGPB as biofertilizers, critically examines their advantages and limitations, and explores how precision biofertilization approaches could improve plant nutrition and crop yields in sustainable agriculture. It further discusses how plant genotypes influence the composition of the PGPB community, which strategies could control and select beneficial microbes, and how mechanistic insights from beneficial plant–microbe interactions could inform complementary crop-improvement strategies, including breeding and genetic engineering, where their biological feasibility, regulatory requirements, and socioeconomic context support their application.

As a narrative review, the literature included and discussed here was selected for its relevance to the major concepts and mechanistic examples addressed, with particular emphasis on plant growth-promoting organisms, beneficial plant–microbe interactions, and the genes, molecular mechanisms, and regulatory processes underlying plant nutrition, growth promotion, and abiotic stress tolerance, as well as the potential application of this knowledge to crop improvement. The review also considers evidence of inconsistent or context-dependent outcomes and distinguishes mechanistic findings from proposed applications where appropriate. As the literature was not selected through a systematic review protocol, the discussion does not represent an exhaustive assessment of all available studies or a quantitative synthesis of effect sizes.

## 2. PGPB: Sustainable, Natural Biofertilizers as an Alternative in Agriculture

Biofertilizers are biological preparations containing beneficial microorganisms. When applied to seeds, plant surfaces, or soil, they colonize the plants and can enhance nutrient availability through various biological mechanisms such as nutrient solubilization and phytohormone production, thereby promoting plant growth and soil health in an environmentally friendly way [20]. The advantages of biofertilizers are evident when compared with conventional organic and chemical fertilizers (Table 1).

Although organic fertilizers such as livestock manure are nutrient-rich and can enhance crop yields, improper management can lead to environmental issues, including soil salt accumulation, nutrient imbalances, pathogen contamination, greenhouse gas emissions, and water pollution from nutrient runoff and leaching [21,22,23]. Chemical fertilizers are also effective at increasing yields, but contribute to soil degradation, nutrient runoff, greenhouse gas emissions, and loss of soil biodiversity, necessitating sustainable alternatives for resilient agricultural systems [24]. Therefore, the application of beneficial microbes as biofertilizers represents a promising approach to sustainable agriculture. Limitations such as inconsistent field performance and poor survival of microbial inoculants may be mitigated by leveraging advances in microbial ecology, formulation technology, and targeted delivery tailored to specific crop and soil requirements [25].

Microbial biofertilizers primarily enhance nutrient availability and uptake rather than replace mineral fertilizers, although biological nitrogen fixation can provide an additional source of nitrogen. They should therefore complement conventional fertilization while accounting for potential ecological risks.

**Table 1:** Comparative characteristics, functions, and limitations of inorganic fertilizers, organic fertilizers, and microbial biofertilizers.

Characteristics	Inorganic Fertilizers	Organic Fertilizers	Microbial Biofertilizers	References
Primary function	Direct supply of mineral nutrients	Nutrient supply through decomposition and mineralization	Biological enhancement of nutrient availability and acquisition	[26]
Nutrient availability	Rapid and readily available	Gradual and mineralization-dependent	Dependent on microbial activity and establishment	[27]
Nutrient concentration and source	High and standardized nutrient concentrations	Lower and variable nutrient concentrations	Living microorganisms rather than concentrated nutrients	[28]
Effects on soil properties	Limited contribution to soil organic matter	Improve soil organic matter and soil properties	Influence rhizosphere processes and nutrient cycling	[29]
Environmental considerations	Excessive use may increase nutrient losses	Promote nutrient recycling but may release nutrients asynchronously	May reduce dependence on mineral fertilizers	[30]
Plant stress-related benefits	Mainly indirect through improved nutrition	Indirect through improved soil properties	Selected PGPB can enhance abiotic stress tolerance	[31]
Consistency of plant response	Generally predictable with appropriate management	Variable with composition, soil, and climate	Variable across strains, hosts, soils, and environments	[32]
Major limitations	Nutrient losses from excessive or inefficient application	Variable composition and nutrient-release rates	Variable survival, establishment, and field efficacy	[33]
Role in sustainable nutrient management	Rapid and controllable nutrient supplementation	Nutrient recycling and soil-quality improvement	Complementary biological tool for nutrient management	[20]

## 3. Key Features of PGPB for Plants

PGPB enhance plant performance under both optimal and stressful conditions through multiple mechanisms. They can increase nutrient availability through the solubilization of phosphorus, potassium, silicon, and zinc, and siderophore-mediated iron acquisition [20]. In addition, certain diazotrophic PGPB can contribute to plant nitrogen nutrition through biological nitrogen fixation, whereas this capacity is not shared by all PGPB [34,35]. PGPB can also produce phytohormones such as auxin (indole-3-acetic acid, IAA) and gibberellins (GAs) that stimulate root growth and plant development [36]. During abiotic and biotic stress, such as drought, salinity, or pathogen infection, these bacteria help plants adjust metabolically and physiologically, modulating stress-responsive pathways and improving resilience by enhancing nutrient uptake, altering hormone balances, and priming host defenses [11].

Endophytic and epiphytic microbes play crucial roles in promoting plant health and productivity. Epiphytic microorganisms can transition into an endophytic lifestyle by colonizing internal plant tissues, and endophytic microbes may reappear on external plant surfaces, resulting in frequent overlap of bacterial species between epiphytic and endophytic niches [37,38]. The major differences between endophytic and epiphytic PGPB include variations in habitat, colonization strategies, plant interactions, functional mechanisms, environmental resilience, practical advantages and limitations, and applications (Table 2).

Endophytic bacteria reside within plant tissues without causing disease and are better protected from environmental factors such as desiccation and soil-borne enemies and competitors than epiphytic bacteria, which colonize external plant surfaces, including the rhizoplane and phylloplane [38]. Endophytes can also form more systemic, metabolically integrated associations with plants that enable nutrient solubilization and phytohormone production [39]. This internal localization may provide endophytes with a relatively protected environment that supports their persistence under stressful conditions. However, their effectiveness as biofertilizer components may be limited by strain specificity, colonization requirements, and host compatibility [40].

**Table 2:** Comparison between endophytic and epiphytic PGPB lifestyles and their relationship to the host plants.

Feature	Endophytic	Epiphytic	References
Habitat/location	Colonize internal plant tissues, including roots, stems, and leaves	Colonize external plant surfaces, including the rhizoplane and phylloplane	[41]
Colonization	Enter and colonize internal tissues via natural openings or wounds	Adhere to the external surfaces of roots or leaves	[42]
Plant interaction	May establish close physiological and metabolic associations with the host	Interact with the host primarily at external plant surfaces	[42]
Main modes of action	Nutrient solubilization, phytohormone production, systemic resistance induction (ISR), and abiotic stress tolerance	Nutrient mobilization, biocontrol through competition, surface pathogen inhibition	[43]
Environmental exposure	Internal localization may provide protection against some external environmental stresses	More directly exposed to environmental fluctuations	[38]
Advantages	Potential for sustained colonization and close interaction with host tissues	Accessible for external application and capable of rapid interactions with plant surfaces	[44]
Limitations	Strain-specificity, complex colonization, potential for incompatibility with the host	Shorter colonization time; reduced efficiency in stressed environments	[45,46]
Application examples	Endophytic consortia for drought tolerance in maize and legumes	Root-surface applied PGPB to enhance phosphate uptake	[47,48]

Recent studies indicate that endophytic communities often exhibit stress-related functional specialization and resilience, particularly under drought, salinity, and disease stress [49]. Endophytes’ internal niche not only shields them from harsh conditions but also facilitates efficient nutrient exchange and phytohormone signaling, making them highly effective biofertilizers and biocontrol candidates [50]. Their ability to persist within host tissues, coupled with reduced competition for nutrients compared to rhizosphere microbes, positions endophytes as promising tools for sustainable crop production under changing climatic conditions [51]. Therefore, endophytic bacteria may offer advantages in persistence and stress tolerance under certain conditions, supporting their potential as complementary tools in sustainable nutrient management and crop protection.

## 4. PGPB-Induced Physiological and Molecular Changes in Plant–Microbe Interactions

### 4.1. PGPB Modulate Phytohormone Balance

Plant–PGPB interactions have been investigated using both conventional experimental approaches and advanced systems-level methodologies, such as multi-omics frameworks (transcriptomics, metabolomics, proteomics, and metagenomics), which are increasingly considered crucial for elucidating the mechanistic foundations of plant–microbe interactions [52,53]. These integrative approaches enable high-resolution characterization of microbially mediated functions in planta, as well as their dynamic modulation across plant developmental stages and varying environmental conditions [54,55].

Phytohormones regulate plant growth, development, and stress responses, and their homeostasis is strongly influenced by PGPB, which can synthesize, degrade, or modulate phytohormones and their signaling networks [56]. Although the products of PGPB have historically been studied for their influence on plants, accumulating evidence indicates a more complex role in coordinating hormonal responses and interactions, nutrient acquisition, and stress-adaptation mechanisms across developmental and environmental contexts by modulating gene expression [57]. For example, research on *Pseudomonas stutzeri* and *Pseudomonas fluorescens* demonstrates their potential to enhance nitrogen uptake in crops such as maize and wheat and to reduce fertilizer dependence via hormone-mediated mechanisms involving auxins and ethylene; however, further studies are needed to clarify their performance and interactions under diverse field conditions for consistent agricultural application [58]. Notably, some endophytic bacterial strains produce stress-related hormones such as abscisic acid (ABA) and jasmonic acid (JA), with increased production under drought, providing direct evidence that microbes can contribute to plant hormonal balance [59]. Therefore, understanding the effect of PGPB on hormonal balance requires an integrative approach and is essential for the effective use of microbial biofertilizers in sustainable agriculture. Transcriptome analysis revealed that *Bacillus velezensis* F21 modulates plant hormone signaling pathways, including ABA, during the induction of systemic resistance (ISR) in watermelon [60]. Additional studies reinforce this multi-dimensional perspective. *Paenibacillus polymyxa* triggered genes in the JA and salicylic acid (SA) pathways in response to the pathogen *Meloidogyne incognita* in a development- and root-zone-specific manner in cucumber, highlighting the importance of spatial and temporal regulation in plant–microbe interactions [61]. *Pseudomonas simiae* altered the expression of the gene encoding the dehydration-responsive element binding protein (*GmDREB2*) in soybean under drought conditions, aligning with ABA biosynthetic pathways and increasing specificity in host–microbe responses [62]. Additionally, the rhizospheric *Bacillus* spp. upregulated *OsPIN1A*, *OsCATA*, and *OsAPX1* in rice under salinity, implying higher auxin transport and antioxidant activities due to inoculation [63]. Similarly, *Enterobacter* sp. SA187 promotes plant growth under low-nitrate conditions by enhancing biomass, root development, and nitrate accumulation via ethylene-dependent regulation of nitrogen uptake and allocation, as evidenced by the loss of these effects in *ein2-1*, *nrt2.5*, and *nrt2.6* mutants in *Arabidopsis thaliana* [64].

Together, these results suggest that PGPB can enhance plant stress resilience by influencing stress-responsive gene expression and transcriptional regulation via interconnected hormonal signaling networks, including auxin, SA, ABA, and JA pathways, which may contribute to stress priming [57]. In addition, some soil microbes trigger induced resistance (IR) in their host plants, thereby positively modulating plant immunity, even in the absence of pathogens. The underlying mechanisms are not yet fully understood, but an elicitor-like mode is likely [65].

### 4.2. PGPB-Mediated Changes in Stress-Induced Gene Expression

In addition to modulating genes involved in phytohormonal homeostasis, PGPB can alter host transcriptional responses associated with stress signaling, defense, osmotic adjustment, antioxidant activity, and development [66]. Transcriptomic and targeted gene-expression studies across different plant–microbe systems have identified changes in transcription factors, stress-protective proteins, antioxidant enzymes, and genes involved in water and hormone regulation under drought, salinity, and low-temperature stress [67,68,69,70,71,72,73]. Representative examples are summarized in Table 3.

Collectively, these studies indicate that beneficial microorganisms can modify host transcriptional networks associated with stress adaptation. However, changes in gene expression following microbial inoculation should be interpreted cautiously, as they may reflect downstream responses to microbial colonization or stress alleviation rather than mechanisms directly responsible for enhanced stress tolerance. Therefore, transcriptional responses are most appropriately viewed as evidence of microbial modulation of host stress-related pathways rather than, in isolation, proof of causal mechanisms.

### 4.3. PGPB Modulate Epigenetic Marks Under Environmental Stress

Recent research shows that rhizosphere microbes not only enhance plant responses to abiotic stress through physiological mechanisms but also can induce epigenetic reprogramming of gene networks responsible for stress adaptation. There is growing evidence that some PGPB can regulate gene expression by modulating epigenetic marks in plants [74]. Epigenetic modifications, including DNA methylation, histone modifications, and small RNA-mediated regulation, have been associated with stress-memory responses that can enhance plant responses to subsequent stress exposure [75].

It was concluded that PGPB can influence epigenetic regulators, such as histone modifications, to activate key genes, such as DREB and HSP, across different plant species under drought stress [76]. For example, inoculation of potato with the endophytic bacterium *Burkholderia phytofirmans* PsJN induces cultivar-specific changes in DNA cytosine methylation patterns, with more pronounced methylation shifts observed in a poorly responsive variety than in a highly responsive one, suggesting differential epigenetic responsiveness among genotypes [77]. Similarly, inoculation of *Phytolacca americana* with *Bacillus* and *Arthrobacter* species reduces global DNA methylation in roots, concomitant with increased transcript abundance and growth promotion [75]. In rice, exogenous sodium butyrate alters rhizosphere sulfur respiration and increases hydrogen sulfide production by plant growth-promoting microorganisms, leading to suppression of the histone deacetylase gene *OsHDA710*, enhanced histone H4 acetylation, and upregulation of ABA- and drought-responsive genes, thereby contributing to improved drought tolerance [78].

An increasing number of studies indicate that microbes can modulate epigenetic regulators, particularly miRNAs, that control stress-responsive pathways [79]. Integrated small-RNA and transcriptome analyses suggest that *Bacillus velezensis* (FZB42) is associated with changes in the expression of maize miRNAs and their predicted target genes, potentially affecting regulators linked to hormone signaling, stress responses, and development, such as pathways linked to oxidative stress tolerance, root architecture, and defense priming, supporting the view that FZB42 functions as a bio-elicitor of host gene expression rather than merely a facilitator of nutrient acquisition [67]. Inoculation of maize with *Azospirillum brasilense* also modifies the expression of multiple miRNAs (including miR159f, miR164a, miR169j, miR396c, and miR399c), thereby reshaping miRNA–mRNA networks that may contribute to stress-related physiological responses such as improved adaptation to abiotic stress and regulation of growth and defense pathways, partially independent of IAA signaling [80]. Additionally, inoculation of maize with *Pseudomonas putida* modulates microRNA expression patterns within distinct zones of the leaf growth region, with several conserved miRNAs, including miR160, miR169, miR319, miR396, and miR408, showing predominantly downregulated expression following bacterial treatment. These changes are accompanied by altered expression of predicted target genes, including *ARF17*, *NF-YA1*, *TCP38*, *GRF15*, and *LAC*, which are implicated in cell division and elongation [81].

Epigenetic regulation has emerged as a central mechanism underlying plant resilience to heat stress, with increasing evidence that beneficial microorganisms can influence these regulatory layers [82]. These epigenetic modifications, including DNA methylation and histone modifications, play key roles in modulating stress-responsive gene expression and developmental plasticity under elevated temperatures. In *A. thaliana*, the root endophyte *Enterobacter* sp. SA187 enhances thermotolerance by increasing H3K4me3 enrichment at heat-stress memory genes, thereby establishing a chromatin state comparable to heat acclimation (priming) [83]. In contrast, as an example of plant-intrinsic epigenetic regulation, histone acetyltransferase TaHAG1 interacts with the transcription factor TaNACL to promote heat stress tolerance in wheat [84], suggesting that histone acetyltransferase-transcription factor (HAT-TF) complexes may modulate chromatin states and stress gene networks in cereal crops.

Importantly, microbe-induced epigenetic changes should not necessarily be interpreted as transgenerational stress memory [85]. Although some beneficial microbes can induce persistent chromatin modifications at stress-memory loci, as demonstrated for *Enterobacter* sp. SA187-mediated H3K4me3 enrichment and thermotolerance [83], persistence within an exposed plant does not establish inheritance through subsequent generations. Evidence for stable transgenerational inheritance of PGPB-induced epigenetic states remains limited, and their persistence after microbial withdrawal and through reproductive development requires further experimental validation. However, associations between microbial inoculation and changes in DNA methylation, histone modifications, miRNA profiles, or gene expression do not, by themselves, establish a causal role in stress tolerance [86,87]. Functional validation using loss- and gain-of-function approaches, reverse genetics, complementation, or targeted manipulation of the implicated regulatory pathways is required to distinguish causal mechanisms from molecular changes that merely accompany microbial colonization or stress responses [88].

### 4.4. Rhizosphere Signaling and Root Exudate–Mediated Interactions

Roots actively trigger specific pathways that reshape the surrounding microbial community, strengthening the plant’s ability to cope with stress [89]. Therefore, exudate reprogramming represents a frontier in synthetic rhizosphere engineering [90,91]. These exudates function not only as nutrient sources but also as signaling molecules that modulate microbial recruitment and community composition [92]. Recent research has illuminated the sophisticated chemical dialogue between plant roots and the rhizosphere microbiome, highlighting root exudates as pivotal mediators of this interaction. For example, certain developmental regulators of *A. thaliana* root hair exudates were recently shown to orchestrate the restructuring of the rhizosphere microbiome under drought, facilitating interactions with beneficial Rhizobiaceae that enhance stress tolerance [93]. Therefore, engineering plants to alter exudate composition under stress could selectively recruit beneficial microbes or mimic microbial signaling molecules internally, activating defense responses without external microbial input [94]. For example, recently, *A. thaliana* was shown to secrete malate under phosphorus (P) limitation, recruiting *Acinetobacter* to mobilize inaccessible P and enhance plant nutrition [95]. Engineering plants for enhanced malate exudation could therefore promote beneficial bacterial recruitment and improve P acquisition in P-deficient soils. Advances in CRISPR/Cas-based gene activation strategies suggest that microbe-responsive regulatory targets can be directly activated in cereal crops to enhance drought recovery traits without reliance on microbial inputs [96]. For instance, CRISPR/Cas-based editing of wheat was used to increase apigenin production, promote free-living nitrogen fixation, and improve yield under nitrogen-deficient conditions, emphasizing that root exudate manipulation has functional impacts even beyond classical legume-rhizobia systems [97]. Manipulation of flavonoid biosynthesis and exudation pathways in *Medicago* sp. profoundly alters rhizosphere microbial composition and nodulation efficiency, particularly under nitrogen-limiting conditions [98]. Similarly, under phosphate-limiting conditions, *Medicago truncatula* modulates flavonoid biosynthesis and exudation via the SPX1/3–PHR2 phosphate-starvation regulatory network, thereby enhancing rhizobial recruitment and activation and promoting nodule formation [99]. However, while research continues to uncover new aspects of these communications, a further challenge lies in distinguishing plant-mediated effects from environmental influences, which often help to shape the microbial community [16].

Beyond the role of exudates, recent advances have illuminated the genetic architecture underlying microbial uptake by plant cells. A landmark study identified a novel cis-regulatory element that facilitated intracellular bacterial uptake by activating symbiosis-related genes in *Lotus japonicus*, even in non-responsive cell types. This *cis*-element was sufficient to enable Nod factor-responsive transcription, highlighting the role of *cis*-regulatory evolution in defining symbiotic capacity [100].

## 5. Learning from PGPB to Reprogram Plants’ Stress Tolerance

The above-described studies suggest that targeted genetic engineering of the host plant could enhance, e.g., nitrogen availability or increase stress tolerance by tuning the underlying signaling and developmental pathways. Thus, the development of genetically modified plants directed by mechanistic knowledge of plant-beneficial microbe interactions is a promising goal. In this context, mechanistic insights from plant–PGPB interactions may identify regulatory targets, metabolic strategies, and stress-responsive processes that can inform crop breeding or genetic engineering. However, microbial functions should not necessarily be regarded as discrete modules that can be transferred directly into plants, because their effects often depend on microbial regulatory networks, community interactions, environmental context, and host–microbe signaling. Available studies provide experimental evidence for several microbe-associated stress-adaptive mechanisms, while others suggest potential targets for plant improvement. Table 4 summarizes these reported microbial effects and their proposed applications, distinguishing experimentally supported findings from strategies requiring further validation.

### Biological Constraints on Translating Microbial Traits into Plants

Translation of microbial stress-adaptation mechanisms into plants must account for the metabolic costs and physiological constraints of heterologous pathway expression [101,102]. Constitutive expression of microbial pathways may divert metabolic resources from plant growth and reproduction, potentially imposing fitness or yield penalties, particularly for multistep pathways with high precursor or energy demands [103,104,105]. Moreover, transgene expression alone does not ensure functional transfer, as microbial enzymes depend on compatible substrates, cofactors, subcellular localization, and host metabolic context. Glycine-betaine engineering illustrates this limitation [106]. Constitutive expression of *Arthrobacter globiformis codA* enabled transgenic *A. thaliana* to synthesize glycine betaine from endogenous choline and enhanced tolerance to salinity and low temperature [107]. In contrast, *Escherichia coli betB* was highly expressed and enzymatically active in transgenic tobacco but could not efficiently support glycine-betaine synthesis because its substrate, betaine aldehyde, was limiting; glycine-betaine production was restored when the substrate was supplied, or its biosynthesis was enabled by the upstream bacterial *betA* gene [108,109]. Thus, successful deployment of microbial traits requires compatibility with host metabolism and, where necessary, appropriate pathway reconstruction and spatial or temporal regulation to balance stress protection against metabolic costs. More broadly, PGPB-mediated stress tolerance is often an emergent property of coordinated microbial metabolism, regulatory networks, host signaling, and interactions among community members rather than the product of a single microbial gene [110]. Consequently, microbial traits should not be considered inherently transferable genetic modules [111]. For example, biological nitrogen fixation requires complex nitrogenase assembly, high energy inputs, and protection from oxygen [112]. Likewise, microbial nutrient mobilization depends on interactions among microorganisms, roots, and soil that cannot be fully replicated by transferring individual genes into plants [113,114]. Therefore, translating microbial traits into crops requires experimental demonstration that the relevant molecular functions remain operative within the regulatory, metabolic, and cellular context of the plant.

**Table 4:** Reported microbial mechanisms and their potential applications for improving plant nutrient acquisition and abiotic stress tolerance.

Mechanism/Focus	Microbial Interaction	Environmental Conditions	Potential Application for Crop Improvement	References
DNA methylation changes by endophytes	*Burkholderia phytofirmans* induces cultivar-specific DNA methylation changes in potato	Drought (general stress context)	Exploiting genotype-specific epigenetic responsiveness in cultivar selection and microbe pairing	[77]
1-aminocyclopropane-1-carboxylic acid (ACC) deaminase activity	PGPB with ACC deaminase lowers plant ethylene by degrading ACC, reducing stress-induced ethylene inhibition of growth.	Drought, salinity, flooding, heavy metals	Points to engineering plants or microbiomes to modulate ethylene levels under stress.	[115]
Rhizosphere signalling and root exudate–mediated interactions	Microbes respond to and shape root exudates, influencing nutrient mobilization, hormone signalling, and stress responses.	Multiple (drought, salinity, nutrient stress)	Supports the design of plants with programmable exudate profiles to recruit beneficial microbiomes.	[97,116]
Reduced global DNA methylation	*Bacillus* and *Arthrobacter* inoculation lowers root DNA methylation in *Phytolacca americana*, increasing transcripts and growth	Drought (general stress context)	Using microbes to fine-tune methylation for growth–stress balance	[75]
Microbiome-mediated histone deacetylase (HDAC) suppression via metabolite-induced H_2_S signaling	Rhizospheric butyrate promotes sulfate-reducing bacteria, increasing H_2_S, which suppresses OsHDA710, enhances H4 acetylation, and activates drought-response genes in rice.	Drought	Engineering rhizosphere metabolite–microbiome axes to modulate HDAC activity and activate stress-associated chromatin programs	[78]
Microbial osmolytes (ectoine, trehalose, glycine betaine)	Biosynthesis genes for ectoine and trehalose in plants enable osmolyte accumulation and osmoprotection.	Drought, salinity, osmotic stress	Shows microbial osmolyte pathways can be engineered into plants, and that regulated or inducible expression can boost tolerance without harming growth.	[117,118,119,120]
miRNA network modulation	*Pseudomonas putida* alters miRNA expression (e.g., miR160, miR169, miR319, miR396, miR408), affecting targets that control cell division/elongation	General stress/growth regulation	Engineering miRNA–target modules to optimize growth under stress	[81]
miRNA-mediated regulation during plant–PGPB interaction	*Azospirillum brasilense* inoculation alters maize miRNA expression and their mRNA targets, partly independent of IAA signaling	General abiotic stress context (growth and stress signaling pathways)	Microbe-driven miRNA reprogramming can modulate growth, hormonal signaling, and stress-response networks to support stress adaptation	[80]
H3K4me3 enrichment and heat memory	*Enterobacter* sp. SA187 increases H3K4me3 at heat-memory loci, mimicking heat priming in *A. thaliana*	Heat	Manipulating heat-memory chromatin marks for durable thermotolerance	[83]
miRNA and transcriptome reprogramming	*Bacillus velezensis* FZB42 alters maize miRNA expression and corresponding mRNA targets enriched in defense and hormone signaling pathways associated with induced ISR	Induced ISR	Using bio-elicitors to modulate host regulatory and defense networks	[67]
Phosphate (P) solubilization	Phosphate-solubilizing bacteria secrete organic acids that mobilize insoluble P, increasing availability to plants.	Nutrient deficiency (P)	Suggests programmable root exudation circuits to release organic acids and mobilize soil phosphorus.	[121]
Siderophore-mediated iron acquisition	Siderophore-producing bacteria chelate Fe^3+^ and increase plant iron uptake under deficiency.	Nutrient deficiency (Fe), often linked to calcareous or alkaline soils	Inspires engineering of plant chelation systems or inducible iron-acquisition pathways.	[122,123]
Malate-mediated recruitment of P-mobilizing bacteria	*A. thaliana*-*Acinetobacter* mutualism	P-deficient soils	Engineering enhanced malate exudation to improve microbial recruitment and P acquisition	[95]

In this regard, for example, microbial osmoprotection pathways offer inspiration. Bacteria accumulate compatible solutes such as trehalose and glycine betaine under osmotic stress [124]. The halotolerant bacterium *Halomonas elongata* confers osmotolerance to tobacco cells by producing the compatible solute ectoine [117]. Transgenic expression of ectoine biosynthesis genes (*ectA*, *ectB*, *ectC*) in cultured tobacco cells enables ectoine accumulation and improves osmotic tolerance [118]. Consistently, transgenic expression of trehalose biosynthesis genes in corn and rice has demonstrated improved drought tolerance [119,120], highlighting the feasibility of transferring microbial osmolyte strategies into plant genomes with controlled expression.

As a translational strategy, microbial nutrient-acquisition mechanisms may inspire complementary plant-engineering approaches. Siderophore-producing bacteria improve plant iron uptake under deficiency [122]. Plants could be engineered with enhanced chelation systems or inducible iron-acquisition pathways modeled on microbial siderophore dynamics [123]. Similarly, phosphate-solubilizing bacteria enhance phosphorus availability by secreting organic acids [121], suggesting that programmable exudation circuits in plants could potentially enhance phosphorus mobilization.

Ethylene exerts dose- and context-dependent control over plant development and stress responses, promoting root growth and differentiation at low levels but suppressing root growth, nodulation, and photosynthetic capacity under stress-induced elevated levels, making it a key target for plant stress engineering [125]. One widely explored biological countermeasure is the use of ACC deaminase–producing rhizobacteria, which cleave ACC, the direct precursor to ethylene, thereby attenuating stress-induced ethylene surges. These bacteria, such as *Pseudomonas fluorescens* and *Enterobacter cloacae*, not only promote root development under abiotic stress but also enhance plant–microbe interactions by alleviating ethylene-induced inhibition of colonization [126,127,128,129]. Introducing synthetic ACC deaminase activity into plants may provide a complementary means of regulating stress-induced ethylene accumulation. In fact, building on microbial interactions, transgenic expression of *acdS*, the bacterial gene encoding ACC deaminase, has been deployed in multiple crops. For example, in rice, canola, and *A. thaliana*, overexpression of *acdS* has significantly improved salt tolerance, water-use efficiency, and protection against oxidative stress, while maintaining root architecture and yield under field conditions [130]. In legumes, ethylene acts as a negative regulator of rhizobial infection and nodule development, and genetically lowering ethylene levels has been shown to increase nodule number and nitrogen-fixation efficiency [131].

## 6. Synthetic Communities (SynCom)

In recent years, besides the application of individual PGPB strains, the deployment of multi-strain microbial consortia as biofertilizers has emerged as a promising strategy to harness functional complementarity. In this approach, microorganisms with complementary functions can be combined to address specific environmental constraints, including nutrient deficiency and abiotic stress. Such consortia may integrate microorganisms involved in nutrient acquisition with those contributing to osmotic adjustment, antioxidant defense, or other stress-protective responses. For instance, Benmrid, et al. [132] demonstrated that a carefully composed bacterial consortium enhanced tolerance to the combined constraints of drought and phosphorus deficiency in legume-cereal intercropping systems through synergistic interactions, illustrating the added value of functional redundancy and diversity. Similarly, in maize, rhizobacterial consortia enriched in members of *Sphingomonas* and Actinobacteria improved drought-related physiological responses, including increased osmolyte accumulation and antioxidant enzyme activity, and were associated with shifts in rhizosphere community composition [133]. Together, these examples illustrate how microbial consortia can combine nutrient-mobilizing and stress-protective functions to address specific environmental constraints. Rational SynCom design requires complementary microbial functions, strain compatibility, and stable root colonization [134]. Although microbial network analyses can identify potential keystone taxa, their functional importance requires experimental validation [135]. Field trials across different soil and climatic conditions are also essential to assess consortium persistence, stability, and effectiveness [136].

However, such benefits of PGPB are often undermined by inter-strain competition and microbial dominance, as native soil microbiota frequently outcompete or inhibit inoculated strains, thereby destabilizing the intended SynCom function [137,138]. Moreover, formulation challenges include maintaining the viability, compatibility, and shelf life of the microbes. Even when consortium members exhibit individual plant growth-promoting traits, their co-formulation often fails due to differential survival or antagonistic interactions during storage and application [139]. These findings underscore that while microbial cocktails theoretically offer advantages over single-strain inoculants, their practical implementation requires refined selection, robust formulation technologies, and systems-level validation under agronomically relevant conditions.

## 7. Precision Agriculture Approaches: Matching Microbiomes to Plant Genotypes

Recently, plant genotype–microbiome compatibility has emerged as a critical factor in inoculant success, yet the contribution of plant genotype to microbiome composition remains less well characterized than that of environmental factors. Obviously, host genetic variation influences root morphology and exudate composition, which in turn shape rhizosphere microbiome assembly and diversity [140]. Evidence from cereal systems indicates that host genotypes play a key role in shaping drought-responsive microbial communities, with potential implications for plant stress physiology under water-limited conditions [141,142].

Precision agriculture approaches enhance the potential of microbiome-plant genotype alignment to improve the specificity and effectiveness of biofertilizers. Through field-scale microbial profiling, researchers can identify microbial taxa closely associated with plant performance under distinct agro-climatic conditions [25]. This has been shown in several cases. For instance, spatial metagenomic analysis across wheat fields has been used to map core rhizosphere microbiota associated with drought adaptation, highlighting spatial heterogeneity in beneficial taxa [143]. Different wheat genotypes recruit distinct rhizosphere microbiomes, and apparently affect growth and disease suppression [144]. The plant host genotype of *Boechera stricta* influences leaf and root microbiome composition in natural environments [145]. The genotype of indica rice also shapes structure and functional potential of the rice rhizosphere microbiome across varieties [146]. Likewise, maize genotype influences root-associated and rhizosphere bacterial community structure and composition in maize cultivars [147]. Similarly, soybean genotypes differentially shape rhizosphere communities, influencing nodulation and nitrogen-fixation efficiency [148].

However, plant genotypes do not always play a major role in reshaping microbial community structure. While in *Brassica napus* the host plant genotype influences the structure of seed-associated microbial communities, environmental factors exert a stronger effect than the host genotype [149], and in date palm, soil salinity strongly restructures both rhizosphere bacterial and fungal communities, although to different levels [150,151]. Similarly, plant genotype had no significant effect on sorghum root microbial community composition, which was driven mainly by location (soil, rhizosphere, root endosphere), time, or drought treatment [152].

Conversely, PGPB application has different effects on plant genotypes. For example, inoculating paddy rice with *Azospirillum brasilense* and *Pseudomonas fluorescens* showed genotype-dependent effects on plants [153]. Consistently, maize hybrids differed significantly in their responsiveness to *A. brasilense* under nitrogen stress, with affecting root traits [154]. Similarly, field experiments tested multiple wheat genotypes and reported genotype-dependent responses to *A. brasilense* inoculation [155]. Plant growth-promoting microbes from wild relatives can shape chrysanthemum root microbiomes and promote growth in a cultivar-dependent manner, with complex arbuscular mycorrhizal fungi communities producing stronger microbiome shifts and greater growth benefits than single-strain inoculants [156]. Precision-agriculture technologies, including soil sensors, remote sensing, and geospatial mapping, could support site-specific microbial inoculant selection and variable-rate application [157]. However, their effectiveness in improving inoculant establishment and crop performance requires more field validation.

**Figure 2:**
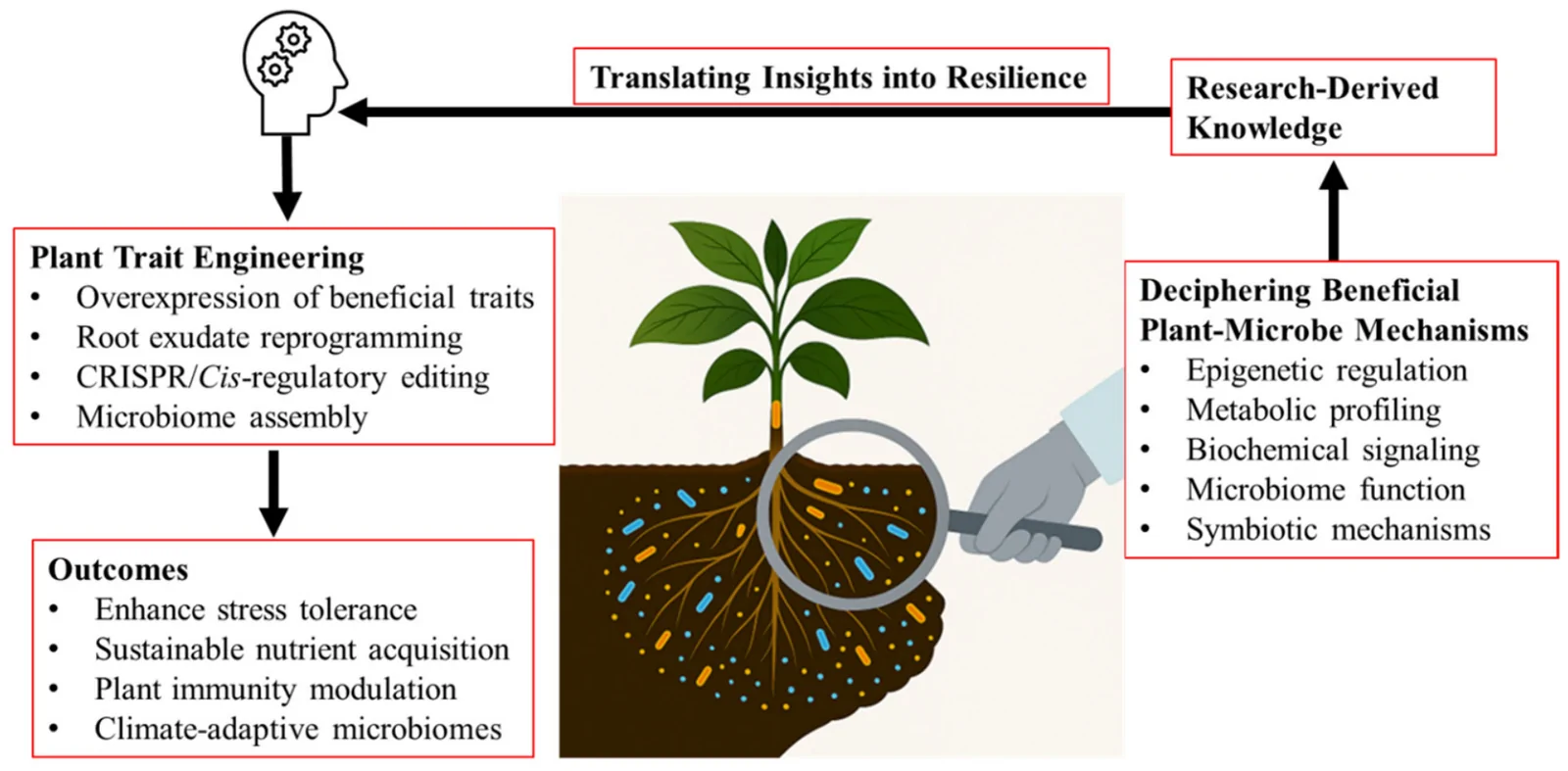
A conceptual framework illustrating mechanism-based engineering of plant–microbiome systems for resilient crop productivity.

Taken together, recent studies clarify that plant growth-promoting microorganisms reshape plant molecular, physiological, and regulatory networks, strengthening resilience to both biotic and abiotic stresses. Further mechanistic insights from these interactions can guide the identification of compounds, key genes, pathways, and regulatory nodes underlying stress tolerance. Such knowledge provides a foundation for precision breeding and genetic engineering strategies to incorporate these resilience traits into plant genomes, with the goal of improving stress tolerance and growth independent of continued PGPB application (Figure 2).

## 8. Barriers to the Use of Biofertilizers

Despite increasing enthusiasm for biofertilizer technologies, their standardization and consistent field efficacy remain elusive due to multifactorial ecological barriers. A significant challenge is soil heterogeneity, which disrupts microbial establishment and leads to spatially variable plant responses. For example, Jansson, et al. [158] found that physical irregularities in soil structure and moisture content altered the colonization potential of introduced microbes, thereby reducing the efficacy of engineered plant-microbiome systems. Soil-borne agricultural pollutants also affect the functioning of PGPB. For instance, herbicide-treated soils drastically alter microbial community composition in maize, resulting in poor persistence of PGPB inoculants [159]. In addition, the use of microbial inoculants in agriculture poses significant challenges for standardization due to their complex, context-dependent interactions with plant and soil environments. Inoculant performance often varies with soil conditions and plant immune responses, leading to inconsistent outcomes [160]. Additionally, the introduction of non-native microorganisms raises ecological and biosafety concerns regarding their persistence and potential effects on native soil microbial communities [19,161]. These issues highlight the need for rigorous evaluation and regulatory frameworks, which currently limit the development of reliable, standardized microbial products.

Furthermore, promising experiments with constitutive expression of selected stress-related genes may present unintended physiological trade-offs, such as interference with reproductive signaling or pathogen defenses, as in the case of ACC deaminase [162]. Consequently, recent strategies emphasize tissue-specific or inducible expression, reducing ethylene levels only during stress episodes or in root-specific zones [163]. This approach preserves ethylene’s developmental roles while optimizing its stress-modulating functions.

Collectively, these challenges require integrated solutions, including site-specific inoculant selection to address soil heterogeneity, compatible microbial consortia and improved formulations to enhance establishment and persistence, and field validation across different soils and host genotypes to assess performance consistency. At the same time, this complexity offers opportunities to refine and tailor microbial applications. Moreover, it must be ensured that crop yields remain high for all treatments. To support and enhance crop-specific biofertilizer design, machine learning models were recently trained on soil and plant genomic data to predict optimal microbial consortia for maize across diverse soil types [164].

Genetic and genome-editing approaches also face translational barriers, including divergent regulatory frameworks, limited technical and biosafety capacity, intellectual-property constraints, and variable public acceptance [165]. These limitations may be particularly important in resource-limited regions, where access also depends on effective breeding and seed-delivery systems. Thus, genetic engineering should be considered complementary to, rather than a universal replacement for, microbial biofertilizers and conventional breeding.

## 9. Conclusions

The interactions between beneficial microorganisms and plants promote plant growth under both optimal conditions and stress, and provide a framework for understanding the mechanisms that govern plant performance and adaptability. Molecular and physiological insights into these interactions can inform microbiome-based strategies and genetic interventions in crop plants, thereby enabling the development of resilient, high-performing plant systems under increasing environmental pressure. The further development of sustainable strategies and concepts requires a shift away from the empirical use of microorganisms and microbial products towards a mechanism-based approach. To this end, the molecular basis of the specific plant-growth-promoting properties of beneficial microorganisms, such as nutrient mobilization, signal transduction, and stress reduction, must be identified.

Translation of these mechanisms into agricultural practice can follow complementary routes. Microbiome-based approaches, including selected PGPB and SynComs, can exploit functional interactions among microorganisms, whereas breeding, genome editing, and synthetic biology may use mechanistic knowledge derived from plant–microbe interactions to modify specific plant traits. However, microbial functions should not be regarded as universally transferable genetic modules, because their effects can depend on microbial regulatory networks, community interactions, host genotype, and environmental conditions. Future progress will therefore depend on integrating mechanistic studies with rigorous functional validation and field testing. Priority research should focus on validating SynCom performance across field environments, integrating microbiome profiling with site-specific agricultural management, and overcoming formulation, regulatory, and economic barriers to practical implementation. Greater attention should be given to identifying microbial functions that remain effective across variable soils and climates, defining compatible and functionally complementary microbial consortia, and determining which microbe-associated mechanisms can be translated into plant improvement without compromising growth or productivity. Combining microbiome management with crop genetics and precision agricultural approaches may ultimately provide more robust strategies for improving nutrient-use efficiency and stress resilience. Rather than replacing microbial approaches with plant engineering, these strategies should be viewed as complementary components of sustainable crop improvement.

## Figures and Tables

**Table 3:** PGPB- and beneficial microorganism-associated changes in plant gene expression under environmental stress.

Beneficial Microorganism	Plant	Stress	Genes or Gene Groups Affected	Reported Expression Response	Functional Association	Reference
*Bacillus velezensis* FZB42	Maize (*Zea mays*)	Biotic stress	Multiple defense- and hormone-associated regulatory genes	Differentially expressed	Induced systemic resistance and stress signaling	[65]
*Bacillus subtilis* B26	*Brachypodium distachyon*	Drought	Drought-responsive genes	Altered expression	Drought response and stress adaptation	[68]
*Bacillus amyloliquefaciens* NBRISN13	Rice (*Oryza sativa*)	Salinity	Genes associated with osmotic regulation, reactive oxygen species (ROS) scavenging, and hormone signaling	Differentially expressed	Osmotic adjustment, antioxidant defense, and hormonal regulation	[69]
*Bacillus amyloliquefaciens* SN13	Rice (*Oryza sativa*)	Abiotic	WRKY, NAC, bZIP, LEA proteins, dehydrins, glutathione S-transferases	Modulated expression	Transcriptional regulation, osmoprotection, dehydration response, and antioxidant defense	[69,70]
*Paenibacillus lentimorbus* B-30488	Chickpea (*Cicer arietinum*)	Drought	Dehydrin 1, lipid transfer protein, prolyl-4-hydroxylase	Increased expression	Osmotic adjustment and drought-associated protection	[71]
*Burkholderia phytofirmans* PsJN	*Arabidopsis thaliana*	Cold, freezing	Cold-responsive genes	Modulated expression	Cold acclimation and improved performance at low temperature	[72]
Arbuscular mycorrhizal fungi	Grape *Vitis vinifera*	Drought	VvNCED, VvP5CS, VvSIP, VvPIP1;2, VvTIP2;1	Upregulated	ABA-associated response, osmotic adjustment, proline metabolism, and water transport	[73]

## Data Availability

No new data were created or analyzed in this study. Data sharing is not applicable to this article.
